# C-755-04. Impact of Targeted Interventions on Hypothermia Events in Burn Patients: A Two-Year Retrospective Review

**DOI:** 10.1093/jbcr/irag033.040

**Published:** 2026-04-06

**Authors:** Vitina Kammin, Viviana Rubio

**Affiliations:** HCA Florida Kendall Hospital, Miami, FL; HCA Florida Kendall Hospital, Miami, FL

## Abstract

**Introduction:**

Hypothermia is a significant risk factor in burn patients due to impaired skin barrier function, increased evaporative heat loss, and frequent operative interventions. Even brief episodes are associated with increased morbidity, mortality, and resource utilization. In early 2024, an unexpected increase in hypothermia events was identified within our verified burn center, prompting a comprehensive review and targeted quality improvement interventions.

**Methods:**

A retrospective review was conducted of all hypothermia events in burn patients between January 2024 and September 2025 across the Burn ICU, Burn OR, and Pre-Operative settings. Hypothermia was defined according to institutional performance improvement (PI) thresholds. After identifying a 2024 hypothermia rate of 7.15% (29 events), a multidisciplinary team initiated a stepwise intervention strategy:

- Education: Targeted thermoregulation education for nursing and surgical staff in the Burn ICU, OR, and Pre-Op areas (January, June/July, and November 2024).
- Equipment & Environment: Additional purchase of blanket warmers, optimization of heated ceiling tiles in Burn ICU, esophageal warmer education, and conversion of the Burn OR PACU into a dedicated pre-op space to minimize exposure risk and promote normothermia.
- Process & Oversight: Daily temperature audits by the Burn PI nurse of all burn inpatients, vital sign validation each shift by Clinical Nurse Coordinators in BICU, and inclusion of burn-specific thermoregulation checkpoints into the OR safety checklist; and monthly compliance & audit tool was also instituted in OR to sustain adherence reported monthly in operations committee meetings.

**Results:**

In 2024, 29 hypothermia events occurred across all care areas. Following initial interventions, the hypothermia rate declined to 2.9% (January 2025) with a few months at 0% and 3.4% (May 2025). After implementation of enhanced monitoring and workflow redesign, no hypothermia events were recorded over the subsequent five months.

**Conclusions:**

A structured, multidisciplinary quality improvement initiative led to sustained elimination of hypothermia events in burn patients following a peak rate of 7.15%. Critical elements included education, environmental modifications, systematic monitoring, and integration of burn-specific practices into existing workflows. Ongoing surveillance and reinforcement of thermoregulation standards remain essential to ensuring patient safety and maintaining gains in care quality.

**Applicability of Research to Practice:**

Structured, multidisciplinary performance improvement initiatives can significantly reduce hypothermia in burn patients. The interventions that were implemented are low cost, reproducible, and feasible across other burn centers and surgical settings. The initiative highlights the value of ongoing surveillance, frontline staff engagement, and sustained compliance monitoring to maintain gains.

**Funding for the study:**

N/A.

